# Compliance with oral nutritional supplements and its influencing factors in postoperative patients with digestive tract tumors: a cross-sectional study

**DOI:** 10.1186/s12912-024-02010-y

**Published:** 2024-06-05

**Authors:** Liqing Su, Jie Zhang, Lei Jia, Wenyue Dou, Mengxue Li, Yumeng Zhang, Jian Chang

**Affiliations:** 1 School of Nursing, Shanghai Jiao Tong University, Shanghai, China; 2 Department of Nursing, Shanghai General Hospital, Shanghai, China

**Keywords:** Digestive tract tumors, Oral nutritional supplements, Compliance, Influencing factor analysis

## Abstract

**Background:**

Oral nutritional supplements are one of the preferred methods of nutritional support for postoperative patients. This study aims to investigate the current status of oral nutritional supplements compliance in postoperative patients with digestive tract tumors and its influencing factors.

**Methods:**

Convenience sampling was employed to select 242 patients who underwent surgery for digestive tract tumors at a tertiary hospital in Shanghai from October 2022 to July 2023 as the study subjects. Data following a normal distribution were analyzed using independent sample t-tests, ANOVA single-factor analysis, Pearson correlation analysis, and multiple linear regression analysis to determine the factors influencing compliance with oral nutritional supplements.

**Results:**

A total of 252 questionnaires were distributed, with 10 invalid questionnaires excluded, resulting in an effective questionnaire rate of 96.03%. The compliance score for oral nutritional supplements in postoperative patients with digestive tract tumors was (2.40 ± 1.45), General Self-efficacy Scale (GSES) score was (24.72 ± 4.86), Multidimensional Scale of Perceived Social Support Scale (MSPSS) score was (58.67 ± 11.09), and Belief about Medicines Questionnaire Scale (BMQ) score was (0.17 ± 2.78). Multiple linear regression analysis revealed that age, adverse reactions, educational level, self-efficacy, medication beliefs, and social support were factors influencing compliance with oral nutritional supplements in postoperative patients with digestive tract tumors (*P* < 0.05).

**Conclusion:**

Our study revealed that the compliance to oral nutritional supplements among postoperative patients with digestive tract tumors was at a moderate level and was closely associated with age, educational level, adverse reactions to oral nutritional supplements, medication beliefs, social support, and self-efficacy. Nursing staff should conduct nursing assessments based on the specific circumstances of patients and their families, provide personalized health education management plans based on the patients’ educational level, enhance patients’ nutrition knowledge, improve patient self-efficacy, and enhance social support for patients, while further improving patient nutrition management.

## Background

Digestive tract tumors refer to tumors that occur in the gastrointestinal tract, primarily including esophageal cancer, liver cancer, gastric cancer, and colorectal cancer, among others. Among these, liver cancer, colon cancer, rectal cancer, and gastric cancer are the most common [1, 2]. As the global incidence of digestive tract tumors continues to rise rapidly, gastrointestinal malignancies account for 23.4% of all new cancer diagnoses worldwide, with a mortality rate of 35.6% [2]. An epidemiological survey revealed that the incidence and mortality rates of digestive tract tumors in China account for 38.71% and 45.03% of the global total, respectively, surpassing the global average [3]. Cancer treatment modalities include medical therapy, surgical intervention, and multidisciplinary collaboration [4]. In recent years, the number of patients undergoing surgical procedures has been steadily increasing [5], necessitating preoperative and postoperative fasting. Furthermore, cancer is a high-metabolism condition, and patients afflicted with cancer often experience poor emotional states, which can lead to reduced food intake [2]. These factors frequently result in patients suffering from malnutrition. Malnutrition is known to adversely affect the prognosis of postoperative cancer patients [6], with studies indicating that patients at nutritional risk often experience compromised clinical outcomes, such as prolonged hospital stays, decreased chemotherapy tolerance, and reduced quality of life [7, 8]. Consequently, improving the nutritional status of postoperative patients has become a focal point of discussion within the field of nutrition.

Clinical guidelines recommend routine nutritional risk screening for all hospitalized cancer patients, followed by nutritional interventions based on the screening results [9]. Current nutritional interventions encompass dietary counseling, Oral Nutritional Supplements (ONS), enteral nutrition, and parenteral nutrition [10]. The European Society for Clinical Nutrition and Metabolism (ESPEN) and multiple guidelines and expert consensus statements recommend ONS as the preferred nutritional support method for malnourished patients [11]. Researches have demonstrated that ONS plays a pivotal role in improving patients’ nutritional status, reducing hospitalization duration, and cutting down healthcare costs [12, 13]. The effectiveness of ONS, however, is contingent upon patient compliance [14].

Compliance refers to the extent to which an individual’s actions align with healthcare provider recommendations concerning medication, diet, and/or lifestyle changes [15]. Compliance with ONS in postoperative patients with digestive tract tumors varies among different countries and regions but generally remains at a low level. In Athens, Lidoriki Irene’s research [14] had assessed the compliance with ONS in malnourished postoperative upper gastrointestinal cancer patients one month after discharge, with results indicating that only 35.9% of patients followed the prescribed ONS regimen. A prospective, multicenter observational study [16] in Spain reported a compliance rate of 78.8% among patients with ONS, consistent with the findings of a previous systematic review, possibly attributed to the high energy and protein content of the provided ONS formulations [17]. Seguy’s research [18] findings indicated that the average compliance rate of patients receiving ONS intervention (ONS with energy > 400 kcal/d, protein > 30 g/d) can reach 83.5%.In Japan, Daisuke’s research [2] has 3-month follow-up study of postoperative patients indicated a decline in compliance from 99.4% preoperatively to 52.7% postoperatively. A study [19] conducted by Ida S and colleagues compared the nutritional outcomes of gastric cancer patients receiving immune-modulating during the perioperative period to those on a standard diet. The study revealed that patients had a 100% compliance rate with ONS before the perioperative period, but this compliance dropped to 50% after the surgery. These findings are consistent with the results obtained by Kong [20] in South Korea. In China, a study conducted by Qin [21] reported varying levels of compliance among patients, ranging from 28.00 to 89.99%.

Compliance is influenced by multiple factors, including factors related to the formulation itself, environmental factors, educational level, and other factors [14, 22–25]. Existing studies on ONS compliance have mainly focused on the investigation of one or a few factors, with limited research exploring the impact of psychosocial factors on compliance. This study aims to investigate the level of ONS compliance in postoperative patients with digestive tract tumors and analyze its influencing factors. It seeks to provide a reference for future personalized nutrition education for postoperative patients, with the ultimate goal of improving patient compliance.

## Materials and methods

### Study design

This cross-sectional study was conducted in a tertiary hospital in Shanghai, China, from September 30,2022 to July 30,2023. Convenience sampling was employed in this study.

### Participants

The subjects of this study were patients confirmed to have digestive tract tumors and undergoing surgical treatment.

Inclusion criteria:1) Patients diagnosed with digestive tract tumors through pathological examination (The clinical diagnoses include colon tumor, rectal tumor, gastric tumor, bile duct tumor, hepatic hemangioma, and pancreatic tumor). 2) Age ≥ 18 years. 3)Postoperative prescription of ONS. 4) Adequate understanding and communication abilities. 5)Willingness to participate in the study and signing the informed consent form.

Exclusion criteria:1) Patients with a history of mental illness, psychiatric disorders, or current psychological conditions. 2)Those with severe physical illnesses that prevent cooperation. 3)Individuals with cognitive impairments or severe hearing impairments.

4)Patients with allergies to milk or protein. 5) Those who do not consent to participate in the study.

### Sample size

Based on the sample size calculation for a cross-sectional study, the sample size should be 5–10 times the number of independent variables [26], with a consideration of a 15% dropout rate. After a literature review, it was determined that there was a total of 16 independent variables, requiring at least 92–184 cases. The study ultimately obtained an effective sample size of 242, meeting the sample size requirements.

### Questionnaire

#### Demographic questionnaire

The questionnaire has been designed based on a review of the literature and consultation with experts. It includes information on the following aspects: age, gender, marital status, educational level, monthly income, employment status, living arrangements, healthcare payment methods, Body Mass Index (BMI), and types of ONS.

#### Morisky medication adherence scale-4 (MMAS-4)

The questionnaire used in this study is the self-reported Morisky Medication Adherence Scale, which was originally developed by Morisky in 1986 [27]. In this study, we used the Chinese version to assess patient compliance to ONS [28]. It consisted of four items. Each item had two response options: “Yes” and “No.” A score of 1 is assigned for “Yes” responses, while a score of 0 is given for “No” responses. A higher score indicates poorer adherence. In this study, the Cronbach’s α coefficient for this questionnaire was calculated to be 0.746.

#### Multidimensional scale of perceived social support (MSPSS)

The scale used in this study is the Multidimensional Scale of Perceived Social Support scale, which was developed by Zimet [29] to assess patients’ perceptions of their social support. This scale evaluates the level of social support perceived by patients from three sources: family, friends, and significant others. The scale consists of 12 items and utilizes a 7-point Likert scale for scoring, where higher scores indicate a higher perceived level of social support. In this study, the Chinese version of the scale was employed [30]. The Cronbach’s α coefficient for this scale in the current study was calculated to be 0.896,

#### Beliefs about medical questionnaire (BMQ)

The Beliefs about Medical Questionnaire Scale was used in this study to assess patients’ beliefs about medication. This scale consists of 10 items and assesses two dimensions: medication necessity and medication concerns. It utilizes a 5-point Likert scale for scoring. The total medication belief score is calculated as the difference between the necessity and concerns dimensions, with a larger difference indicating a more positive belief in medication. The Chinese version of the scale demonstrates good internal consistency [31]. In this study, the Cronbach’s α coefficient for the questionnaire was calculated to be 0.703.

#### General self-efficacy scale (GSES)

This study employed the General Self-Efficacy Scale to assess the self-efficacy of the research subjects. The General Self-Efficacy Scale used in this study was developed by Schwarzer [32]. It is available in a Chinese version and comprises a total of 10 items [33]. The scale uses a 4-point Likert rating method, with scores ranging from 10 to 40 points. A higher score indicates a higher level of self-efficacy perceived by the patients. In this study, the Cronbach’s α coefficient for the scale was calculated to be 0.872.

### Methods for data collection

Patient general information was collected through the hospital’s medical records system. One month after the prescription was issued, when the patients returned to the hospital outpatient clinic for a follow-up visit, face-to-face data collection was conducted. The researcher used a standardized language to explain the questionnaire’s completion requirements to the patients and obtained their informed consent by signing an informed consent form. If a patient was unable to fill out the questionnaire independently, the researcher assisted the patient by asking questions and recording their responses. At the conclusion of the survey, the researcher immediately checked the completeness of the questionnaire. In cases of missing information, the researcher promptly asked the patient and filled in the missing details. A total of 252 questionnaires were distributed in this survey, with 10 incomplete ones excluded. A total of 242 valid questionnaires were collected, resulting in a questionnaire validity rate of 96.03%.

### Statistical analysis

Data were double-entered using Epidata 3.1 software and then processed and analyzed using SPSS 25.0. Descriptive statistics were used to represent continuous data as mean ± standard deviation(M ± SD) and categorical data as proportions and frequencies. Independent sample t-tests and one-way ANOVA were employed for the analysis of categorical variables, while Pearson correlation analysis was used for continuous variables. Multiple linear regression analysis was conducted for multifactor analysis, with significance levels set at *P* < 0.05 or *P* < 0.01 to indicate statistical significance.

## Results

### Characteristics of the study sample

This study ultimately included 242 postoperative patients with digestive tract tumors. The patients had an average age of (64.82 ± 12.92) years, and their average BMI was (23.49 ± 3.44) kg/m^2^. All patients in the study used powdered ONS (100%). General information about the patients can be found in Table 1.

### Current situation analysis of compliance of ONS

The compliance score for ONS in postoperative patients with digestive tract tumors was (2.40 ± 1.45), BMQ scale score was (0.17 ± 2.78), the GSES score was (24.72 ± 4.86), and MSPSS score was (58.67 ± 11.09).

### Variance analysis of compliance of ONS

The independent samples t-test was conducted for general data, and one-way ANOVA was used for multiple groups. The statistical test results are presented in Table 1. The results indicate that age, education background, monthly income, adverse reactions, and type of disease all have a statistically significant impact on patient compliance (*P* < 0.05). (Table 1)

**Table 1:** General information and univariate analysis of compliance in postoperative patients with digestive tract tumors (*N*=242)

Type		Number (%)	F/t	*P* value
Gender	Male	136(56.2%)	-0.977^a^	0.329
	Female	106(43.8%)		
Age(year)	<65	113(46.7%)	-0.593^a^	<0.001
	≥65	129(53.3%)		
BMI (kg/m^2^)	<24	136(56.2%)	-0.559^a^	0.577
	≥24	96(39.7%)		
Marital Status	Single	8(3.3%)	2.341^b^	0.056
	Married	229(94.6%)		
	Divorced	5(2.1%)		
Living Arrangement	Single	8(3.3%)	0.867^b^	0.485
	With spouse	162(66.9%)		
	With Family	70(28.9%)		
	With Others	2(0.8%)		
Education Background	Not educated	8(3.3%)	3.595^b^	0.007
	Primary School	68(28.1%)		
	Middle School	88(36.4%)		
	High School	49(20.2%)		
	College/University	29(12.0%)		
Monthly Income(CNY)	<2000	65(26.9%)	3.166^b^	0.015
	2000∼3000	72(29.8%)		
	3000∼4000	61(25.2%)		
	4000∼5000	28(11.6%)		
	≥5000	16(6.6%)		
Employment Status	Unemployed	28(11.6%)	1.215^b^	0.305
	Employed	44(18.2%)		
	Retired	170(70.2%)		
Healthcare Payment Method	Free Medical Care	39(16.1%)	1.275^b^	0.281
	Employee Insurance	66(27.3%)		
	Urban Medical Insurance	40(16.5%)		
	New Rural Cooperative Medical Scheme	88(36.4%)		
	Out-of-Pocket	9(3.7%)		
Adverse Reaction	Diarrhea	48(19.8%)	5.495^b^	<0.001
	Abdominal Distension	52(21.5%)		
	Nausea	10(4.1%)		
	Vomiting	3(1.2%)		
	None	129(53.3%)		
Type of Disease	Colon Tumor	104(43.0%)	2.649^b^	0.034
	Rectal Tumor	61(25.2%)		
	Gastric Tumor	68(28.1%)		
	Hepatic Hemangioma	7(2.9%)		
	Pancreatic Tumor	1(0.4%)		
	Bile Duct Tumor	1(0.4%)		

*Note* BMI=Body Mass Index, CNY = Chinese Yuan. *t*=independent sample t-test, *F*=One-way ANOVA, a=*t* value, b=*F* value

### Correlation analysis of ONS compliance with social support, medication beliefs, and self-efficacy

The research results show that the compliance score is negatively correlated with the total score of MSPSS, the total score of BMQ, and GSES (*r* = -0.377, -0.250, -0.309, *P* < 0.001). (Table 2).

**Table 2:** Correlation analysis of ONS compliance and influencing factors in postoperative patients with digestive tract tumors (*N* = 242)

Influencing factors	*r* value	*P* value
MSPSS	-0.377	< 0.001
BMQ	-0.250	< 0.001
GSES	-0.309	< 0.001

^§^*p*-value has been calculated using pearson correlation analysis. MSPSS, Multidimensional Scale of Perceived Social Support. BMQ, Beliefs about Medical Questionnaire. GSES, General Self-Efficacy Scale

### Multivariate analysis of ONS compliance in postoperative patients with digestive tract tumors

Multiple Linear Regression with Compliance as the dependent variable was conducted using variables that showed statistically significant differences in the univariate and correlation analyses as independent variables. The values assigned to the independent variables are presented in Table 3.

**Table 3:** Assignment of independent variables in multiple linear regression

Independent variables	Assigning values to variables
Age (Years)	< 65 = 1, ≥ 65 = 2
Education background	Not educated = 1, Primary School = 2, Middle School = 3, High School = 4, College/University = 5
Monthly income (CNY)	< 2000 = 1, 2000 ∼3000 = 2, 3000 ∼;4000 = 3 ,4000 ∼5000 = 4, ≥ 5000 = 5
Adverse reaction	Diarrhea= (0,0,0,0,0), Abdominal Distension= (0,1,0,0,0), Nausea= (0,0,1,0,0), Vomiting= (0,0,0,1,0), None= (0,0,0,0,1)
Type of disease	Colon Tumor= (0,0,0,0,0,0), Rectal Tumor= (0,1,0,0,0,0), Gastric Tumor= (0,0,1,0,0,0), Hepatic Hemangioma= (0,0,0,1,0,0), Pancreatic Tumor= (0,0,0,0,1,0), Bile Duct Tumor= (0,0,0,0,0,1)

*Note* CNY = Chinese Yuan

The total scores of MSPSS, BMQ, and GSES were input as their original values. The results indicate that age, education background , adverse reactions, type of disease, medication beliefs, self-efficacy, and social support are independent influencing factors for patient ONS compliance. (Table 4)

**Table 4:** Multiple linear regression analysis of postoperative patients with digestive tract tumors (*N* = 242)

Independent variables		B	SE	Beta	t	*P* value
(constant)		5.338	0.696		7.673	< 0.001
BMQ		-0.075	0.030	-0.143	-2.479	0.014
GSES		-0.050	0.017	-0.168	-2.959	0.003
MSPSS		-0.028	0.008	-0.213	-3.665	< 0.001
Education background		-0.227	0.080	-0.163	-2.832	0.005
Age (Years)		0.569	0.170	0.196	3.350	0.001
Monthly income (CNY)		-0.002	0.072	-0.002	-0.026	0.979
Type of disease	Rectal Tumor	0.087	0.197	0.026	0.441	0.660
	Gastric Tumor	-0.135	0.191	-0.042	-0.710	0.479
	Hepatic Hemangioma	-0.130	0.478	-0.015	-0.273	0.785
	Pancreatic Tumor	-1.854	1.229	-0.082	-1.509	0.133
	Bile Duct Tumor	1.518	1.228	0.067	1.236	0.218
	Colon Tumor	reference				
Adverse reaction	Abdominal Distension	0.186	0.248	0.053	0.749	0.454
	Nausea	0.722	0.426	0.099	1.694	0.092
	Vomiting	-0.562	0.730	-0.043	-0.770	0.442
	None	-0.477	0.213	-0.164	-2.240	0.026
	Diarrhea	reference				

*Note R* = 0.601, *R*^*2*^ = 0.361, adjust *R*^*2*^ = 0.319, *F* = 8.513, *P* < 0.001. *SE*, Standard Error. CNY, Chinese Yuan. MSPSS, Multidimensional Scale of Perceived Social Support. BMQ, Beliefs about Medical Questionnaire. GSES, General Self-Efficacy Scale

## Discussion

One of our research objectives was to assess the current status of ONS compliance among postoperative patients with digestive tract tumors in southern China. The survey results revealed that the ONS compliance of postoperative patients with digestive tract tumors was at a moderate level, with an average score of 2.40 ± 1.45 points, and it was closely related to age, educational level, adverse reactions, medication beliefs, social support, and self-efficacy.

Regarding the current state of ONS compliance, our survey results were consistent with the findings of Qin and Lidoriki [14, 21]. However, other randomized controlled trials (RCTs) reported results indicating high ONS compliance among patients with gastric or colorectal cancer [16, 34, 35]. The reasons for differences in compliance could be attributed to the “trial effect” commonly seen in clinical trials. Patients tended to have a better understanding of their condition during the intervention process, and healthcare providers used various methods to remind patients to take ONS, thereby encouraging good compliance and maintaining high compliance levels. However, in real-world clinical settings, maintaining good adherence could be challenging. Healthcare providers could use various methods to remind patients to take ONS in order to improve ONS compliance.

Our finding showed that patients’ compliance of ONS was related to age and education background. The older the patients, the lower their compliance. This might have been related to age-related declines in smell and taste [36]. As patients’ memory declined with age, they might have missed taking nutritional supplements, thereby reducing ONS compliance. Patients with a higher level of education were more likely to understand the content of health education provided by healthcare professionals. These patients might have been inclined to recognize the benefits of ONS in enhancing immune function and shortening hospital stays and might have followed medical advice to take ONS formulations, thereby improving their compliance. In the future, personalized health education content could have been developed for patients based on their age and level of education to increase their knowledge and improve compliance.

ONS, as one of the formulations of enteral nutrition [37], commonly led to adverse reactions such as bloating, diarrhea, nausea, and vomiting. The study which conducted by Wang suggested that patients often experienced nausea and vomiting due to the unpleasant taste and low sweetness of ONS. Some studies showed that patients who received low-density, high-energy interventions had higher compliance [38]. Most ONS formulations were high-density and high-energy [39], and the rapid entry of high-density substances into the intestine could have easily led to diarrhea [40]. Additionally, ONS containing high protein and dietary fiber could have produced gas and caused bloating in patients during the process of digestion and absorption in the intestine [41], leading to decreased compliance. In the future, selecting low-density, high-energy foods could have mitigated adverse reactions in patients.

The social support score of patients was 58.67 ± 11.09, which was at a moderate level. Types of social support included support from within the family, support from outside the family (relatives and friends), and support from society (healthcare professionals, etc.). The higher the social support patients received, the higher their compliance [42], and vice versa. Research results had shown that patients who lived with family members or relatives had higher compliance [42]. Patient compliance was also related to health education provided by the medical team, such as doctors, nurses, and nutritionists. Arribas’s [43]study indicated that receiving support from the medical team increased compliance from 41 to 67%, which might have been related to patients perceiving support from healthcare professionals. Therefore, healthcare professionals could have explained the importance of family support to patients’ families, increased both internal and external support from the family, and used multidisciplinary team support to enhance societal support, thereby improving ONS compliance.

The medication belief score of patients was 0.17 ± 2.78, which was at a low level. Medication belief referred to the relationship between patients’ perception of the necessity and concerns about taking medication [44]. In this study, patients’ medication belief was at a low level, indicating that patients had strong concerns about medication. Patients’ concerns about taking ONS mainly manifested in three aspects: patients might have worried about adverse reactions caused by ONS, they might have been concerned about the negative effects of ONS on their underlying conditions, and they might have feared that taking ONS might have reduced the effectiveness of other medications, leading to a decrease in ONS dosage and patient compliance [21]. The reasons for this might have been that patients had limited knowledge of ONS as a form of nutritional support. Traditional nutritional support was provided through nasogastric feeding or peripheral intravenous routes, and there was limited public awareness of nutritional support through ONS, leading to patients having strong concerns about ONS. Combined with adverse reactions to ONS and patients’ consideration of their own interests, this led to a decrease in patients’ medication belief and compliance. In the future, educating patients about the benefits and adverse reactions of ONS could help alleviate their concerns.

The self-efficacy score of patients was 24.72 ± 4.86, which was at a moderate level. The lower the self-efficacy, the worse the compliance of patients, so improving patients’ self-efficacy could have helped enhance their compliance. Self-efficacy referred to patients’ confidence in using their skills to accomplish a task [45]. Researches had shown that patients with higher self-efficacy had higher compliance [46, 47]. The reasons for this might have been that self-efficacy helped patients learn self-management and psychological adjustment, enhance their self-management abilities, and increase their confidence in taking ONS as prescribed by healthcare professionals, thereby improving compliance. In the future, positive encouragement could have been provided to patients to enhance their self-efficacy and improve their self-management abilities, thereby increasing compliance.

### Strengthens

Through exploration of the current status and influencing factors of ONS compliance among postoperative patients with digestive tract tumors in China, we found that ONS compliance among these patients is generally low. Our cross-sectional study revealed that factors influencing good ONS compliance among postoperative patients with digestive tract tumors include age, education background, monthly income, adverse reactions, type of disease, social support, self-efficacy, and medication beliefs. Among these factors, age, adverse reactions, educational background, self-efficacy, social support, and medication beliefs are independent influencing factors of ONS compliance. Previous studies have mainly focused on formulations, age, adverse reactions, and disease types, factors that cannot be easily changed through nursing interventions. There has been limited exploration of ONS compliance from a social psychological perspective. This study supplements previous research by investigating the influence of social psychological factors such as social support, self-efficacy, and medication beliefs on ONS compliance among digestive tract tumors patients.

### Limitations

This study has some limitations. Firstly, it was conducted in a single hospital in Shanghai, and the sample size was relatively small. Future research could benefit from expanding the study to multiple centers to provide a more comprehensive understanding of compliance in different settings.

Secondly, the hospital where data was collected is located in a suburban area, which may have resulted in a lower level of education among the participants. This could have influenced the lower education level observed in the study. Future studies could include data from urban hospitals to provide a broader perspective on patient education levels.

Additionally, this hospital only had powdered ONS available, and there were no solid or liquid formulations. This limited the discussion regarding the impact of different ONS formulations on compliance. Future research could investigate the influence of powdered versus solid ONS formulations on compliance.

Furthermore, there is currently no specific scale for assessing ONS compliance. This study used the Morisky Medication Adherence Questionnaire to collect data on ONS compliance. Future research should consider developing measurement tools tailored to ONS compliance to better address this aspect of patient care.

Overall, these limitations highlight areas for future research and improvement in understanding and enhancing compliance with ONS in postoperative digestive tract tumor patients.

## Conclusion

The compliance with ONS among postoperative digestive tract tumor patients was found to be at a relatively low level in this study. The main influencing factors identified include age, education background, adverse reactions, monthly income, type of disease, social support, medication beliefs, and self-efficacy. These findings suggest that healthcare providers should conduct follow-up visits for each patient, tailor personalized intervention strategies based on individual characteristics, and work towards improving patient compliance with ONS. Ultimately, this can lead to improved nutritional status among these patients.

## Data Availability

The datasets used and analyzed during the current study are available from the corresponding author on reasonable request.

## Abbreviations

*ESPEN*: European Society for Clinical Nutrition and Metabolism
*ONS*: Oral Nutritional Supplements
*BMI*: Body Mass Index
*CNY*: Chinese Yuan
*MMAS-4*: Morisky Medication Adherence Scale-4
*MSPSS*: Multidimensional Scale of Perceived Social Support
*BMQ*: Beliefs about Medical Questionnaire
*GSES*: General Self-Efficacy Scale
